# Lifestyle interventions can reduce the risk of Barrett’s esophagus: a systematic review and meta‐analysis of 62 studies involving 250,157 participants

**DOI:** 10.1002/cam4.4061

**Published:** 2021-06-15

**Authors:** Zhanwei Zhao, Zifang Yin, Chaojun Zhang

**Affiliations:** ^1^ Department of General Surgery the Sixth Medical Center of PLA General Hospital Beijing China; ^2^ Department of Obstetrics the Sixth Medical Center of PLA General Hospital Beijing China

**Keywords:** Barrett's esophagus, esophageal adenocarcinoma, lifestyle, meta‐analysis

## Abstract

**Background:**

Barrett's esophagus (BE) is a well‐established risk factor for esophageal adenocarcinoma. Our objective was to investigate the effectiveness of lifestyle interventions on BE risk.

**Methods:**

We searched PubMed, Embase, and Web of Science up to 30 September 2020. The summary relative risks (RRs) and 95% confidence intervals (CIs) for the highest versus lowest categories of exposure were assessed. Analyses of subgroup, dose–response, sensitivity, and publication bias were conducted.

**Results:**

Sixty‐two studies were included that involved more than 250,157 participants and 22,608 cases. Seven lifestyle factors were investigated: smoking, alcohol, body mass index (BMI), physical activity, sleep time, medication, and diet. We observed statistically significant increased BE risks for smoking (RR = 1.35, 95% CI = 1.16–1.57), alcohol intake (RR = 1.23, 95% CI = 1.13–1.34), body fatness (RR = 1.08, 95% CI = 1.03–1.13), less sleep time (RR = 1.76, 95% CI = 1.24–2.49), and proton pump inhibitors use (RR = 1.64, 95% CI = 1.17–2.29). Reduced risks of BE were found for aspirin (RR = 0.70, 95% CI = 0.58–0.84) and the intake of vitamin C (RR = 0.59, 95% CI = 0.44–0.80), folate (RR = 0.47, 95% CI = 0.31–0.71), and fiber (RR = 0.95, 95% CI = 0.93–0.97). The quality of most included studies was high and the subgroup analysis according to the quality score showed significant results (*p* < 0.05). There was no publication bias for smoking and alcohol. Although the analysis suggested significant evidence of publication bias for BMI, sensitivity analysis showed that the changes in the recalculated RRs were not significant.

**Conclusions:**

The large meta‐analysis revealed that lifestyle modifications could reduce the risks of BE and, consequently, esophageal adenocarcinoma.

## Graphical abstract

We conducted a large meta‐analysis based on sufficient evidence and a quantitative synthesis of the eligible data published up to 30 September 2020, to investigate the effectiveness of lifestyle interventions on Barrett's esophagus risk. In this meta‐analysis of 62 studies that involved more than 250,000 participants and 22,000 cases, we found that smoking, alcohol intake, high levels of body fatness, and less sleep time are associated with Barrett's esophagus risk. There are statistically significant reduced risks of Barrett's esophagus with aspirin use and vitamin C intake, folate, and dietary fiber. In a word, these findings strengthen our understanding of the potential mechanisms of Barrett's esophagus development and highlight awareness that lifestyle intervention could reduce the risks of Barrett's esophagus and, consequently, esophageal adenocarcinoma.

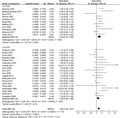

## 1 INTRODUCTION

Esophageal cancer is a highly lethal cancer with 572,000 new cases and 509,000 deaths occurring worldwide in 2018.^1^ Considering the increasing trend in the incidence of and the high fatality of esophageal cancer, finding novel strategies to prevent the development of this cancer is an urgent need. Barrett's esophagus (BE) is considered a well‐established risk factor^2^ and the only known precursor for esophageal adenocarcinoma.^3^ Esophageal adenocarcinoma is estimated to be at least 10 times as high among patients with BE as it was in the general population.^4^

Recently, an increasing number of studies have focused on lifestyle and modifiable risk factors for BE.^5^, ^6^, ^7^, ^8^, ^9^ However, the associations between several factors and BE risk are inconsistent, including alcohol,^10^, ^11^ BMI,^12^, ^13^ and nonsteroidal anti‐inflammatory drug (NSAID) use.^14^, ^15^ Although smoking has been systematically evaluated,^16^ the population included was limited to males. Furthermore, the included studies of that meta‐analysis were published up to 2011. Numerous high‐quality studies have appeared during the last approximately 5 years, and an updated meta‐analysis may clarify the impact of the recent studies. To the best of our knowledge, no other main lifestyle factors have been assessed systematically with respect to BE risk.

Thus, given the large burden of esophageal adenocarcinoma worldwide and the controversial evidence of BE, we conducted a large systematic review and meta‐analysis with the following objectives: (1) to provide an update based on more‐sufficient evidence and a quantitative synthesis of the eligible data on the associations between lifestyle factors and BE risk; (2) to conduct dose–response analyses to further evaluate potential dose–response associations, where possible; and (3) to perform subgroup analyses to further explore the associations by study design, geographic area, publication year, sample size, quality score and adjustments, including smoking, alcohol, BMI, and reflux, where possible.

## 2 METHODS

### 2.1 Selection criteria

The selection criteria were independently judged by two reviewers (ZZ and ZY), which were as follows: we selected the highest quality studies, the largest samples, and the most recent studies for the studies reporting the similar data; given the varied diagnostic criteria of BE,^17^ both of the American College of Gastroenterology clinical guidelines^18^ and the British Society of Gastroenterology guidelines^19^ on the diagnosis of BE met the eligibility criteria; narrative, systematic reviews, and meta‐analysis were excluded if they did not include original data; editorials, letters, comments, case reports, and conference abstracts were excluded; studies in which only the abstract could be obtained were excluded; esophagitis, esophageal cancer, gastrointestinal stromal tumors, and other tumors of the esophagus were excluded; study populations of other comorbidities (e.g., inflammatory bowel disease, adenomas, polyps, and diverticulitis) were excluded; the language of included studies was limited to English; and studies were limited to those involving humans.

### 2.2 Search strategy

We searched PubMed, Embase, and Web of Science to identify studies published from inception through 30 September 2020. Details of the search terms (keywords or Medical Subject Heading terms) were: “lifestyle,” “risk(s),” “diet/dietary,” “food(s),” “smoking/smoker/tobacco/cigarette(s),” “drink/drinking/alcohol/ethanol/alcoholic/beverage(s)/wine/beer/spirits/liquor,” “fatness/obesity/obese/obeseness/adiposity/overweight/weight/body mass index/BMI/waist hip ratio/waist circumference/hip circumference,” “physical activity/exercise,” “sleep/nocturnal,” “medicine/medical/medication(s),” “nonsteroidal anti‐inflammatory drug(s)/NSAID(s)/ibuprofen/diclofenac/naproxen/indomethacin/mefenamic acid/piroxicam/ketoprofen/etodolac/meloxicam/rofecoxib/flurbiprofen/phenylbutazone/aspirin,” “proton pump inhibitor(s)/PPI(s)/omeprazole/pantoprazole/esomeprazole/lansoprazole/dexlansoprazole,” “statin(s)/hydroxymethylglutaryl‐CoA reductase inhibitor/simvastatin/lovastatin,” “nutrition/nutrient,” “vitamin(s),” “folate/folic acid,” “fiber(s)/fibre(s),” “meat(s)/fish/poultry/chicken/turkey/duck,” and “selenium” in combination with “Barrett's/Barrett,” “esophagus/oesophagus/esophageal/oesophageal/neoplasia/neoplasm/neoplasms,” The two sets of keywords were combined individually, and the eligibility criteria were independently judged by two reviewers (ZZ and ZY).

### 2.3 Study quality and data extraction

The study quality of cohort studies and case–control studies was assessed using the Newcastle–Ottawa Scale (NOS).^20^ The NOS range is 0–9 stars, and a high‐quality study includes 7 or more stars. The NOS is judged on three factors, including the elucidation of the exposure or outcomes of interest, the selection of the study populations, and the comparability of the populations. An 11‐item checklist recommended by the Agency for Healthcare Research and Quality (AHRQ) was used to assess the methodological quality of cross‐sectional studies. The range of AHRQ is 0–11 scores. Low quality is 0–3, moderate quality is 4–7, and a high‐quality study ranges from 8 to 11. Two reviewers (ZZ and ZY) independently assessed the study quality, and discrepancies in interpretation were resolved by a consensus decision made by the third reviewer (CZ).

A data extraction sheet was generated for each study. Detailed information included the first author, publication year, country, study type, study period, study population, assessment method, type of exposure measured, exposure categories, adjusted RR (95% CI), adjusted variables, and quality score.

### 2.4 Statistical analysis

SPSS 22.0 (Chicago, Illinois, USA) was used to collect and extract data. RevMan5.3 (The Cochrane Collaboration, Oxford, UK) software was used for the synthesis and analysis of data based on relative risks (RRs) and 95% confidence intervals (95% CIs).

We conducted this meta‐analysis for the risk of BE and smoking, BMI, physical activity, sleep duration, medications, and dietary factors. Medications included aspirin, NSAIDs, PPIs, and statins. Dietary factors included alcohol, vitamin C, folate, selenium, total meat, and white meat. Stratified analysis was not performed for alcohol. Beer, wine, and spirits were included. Fruits, vegetables, fat, red meat, and processed meat were excluded because we have previously analyzed these issues.^21^ Vitamin D and calcium were excluded due to the limited studies. A random‐effects model was used to pool the RRs and 95% CIs if there was heterogeneity among studies, and a fixed‐effects model was used if there was no heterogeneity. The method described by Greenland et al^22^ was used for the nonlinear dose–response analysis. Studies that reported at least three quantitative exposure categories for RRs with their corresponding 95% CIs were included for dose–response analysis.

Heterogeneity among studies was detected using *I^2^* statistics (*I^2^* < 50% was considered low heterogeneity, and *I^2^* > 50% was considered to indicate substantial heterogeneity)^23^ and Q statistics (*p* < 0.1 was considered representative of significant heterogeneity). Gastroesophageal reflux disease (GERD) is a well‐established risk factor for the development of BE.^24^ The data of non‐GERD patients as the control group were preferred for summary estimates to eliminate possible heterogeneity. Additionally, a subgroup analysis was conducted to further explore the sources of heterogeneity by study design, geographic area, publication year, sample size, quality score, and adjustments (smoking, alcohol, BMI, and reflux symptom), where possible.

Publication bias was assessed using funnel plots and Egger's test (*p* < 0.1 was considered significant publication bias).^25^ Sensitivity analyses were conducted by removing one study at a time to investigate the influence of a specific study on the pooled risk estimate.

## 3 RESULTS

### 3.1 Literature selection, study characteristics, and quality scores

Figure S1 shows the flowchart of the search strategy for selecting the eligible studies. In total, 5712 studies were initially identified; 5079 studies were excluded for duplication and 633 studies were selected for further consideration after excluding the duplicates deriving from individually combination of search terms. Of those, 529 studies were excluded after reviewing the titles and abstracts, and 56 studies were included after reviewing the full‐text article. Finally, 62 studies met the eligibility criteria after including 6 studies from the reference review.

The 62 selected studies were conducted in 16 countries or regions worldwide and involved more than 250,157 participants and 22,608 cases. These included studies provided 128 separate estimates to the associations of lifestyle factors and BE risk. More detailed information on these studies has been listed in Table 1.

**TABLE 1:** Baseline characteristics of included studies for lifestyle factors and Barrett's esophagus risk

First author, year, country	Study design	Study/institution period	Case/control (cohort, n)	Type of exposure	Exposure categories	Adjusted RRs (95% CIs)	Adjusted variables	Quality score
Akiyama 2009 Japan^26^	CO	The Gastroenterology Division of Yokohama City University Hospital 2005–2006	374/869	Smoking Alcohol BMI	Current versus no Yes versus no 25.8 versus 24.1 kg/m^2^	1.92 (1.36–2.70) 1.23 (0.86–1.76) 1.02 (0.98–1.07)	Age, sex, BMI, drinking, gastric mucosal atrophy, and erosive esophagitis	7
Anderson 2006 Northern Ireland and the Republic of Ireland^27^	CC	The FINBAR study 2002–2004	224/260	NSAID Aspirin	Yes versus no at or before 5 year versus never	0.49 (0.22–1.09) 0.69 (0.38–1.26)	Age, sex, education, job type, smoking, alcohol, BMI, location, GERD, hiatus hernia, peptic ulcers, and esophagitis	7
Anderson 2007 Northern Ireland and the Republic of Ireland^28^	CC	The FINBAR study 2002–2004	224/260	Smoking BMI	Current versus no >29 versus <25.8 kg/m^2^	1.41 (0.77–2.58) 0.75 (0.44–1.25)	Age, education, job type, and GERD	7
Anderson 2009 Northern Ireland and the Republic of Ireland^29^	CC	The FINBAR study 2002–2004	224/260	Alcohol	>39.7 g/week versus never	0.77 (0.40–1.51)	Age, sex, smoking, job type, education, energy, fruits and vegetables, *H pylori* infection, NSAIDs, and GERD and location	8
Avidan 2001 USA^30^	CC	The Department of Veterans Affairs (VA) Hospital in Hines, Illinois 1979–1996	1016/3047	Smoking Alcohol	Current versus no Yes versus no	0.92 (0.77–1.10) 1.31 (1.11–1.55)	Age, male gender, alcohol, hiatus hernia, and gastric surgery	7
Balasubramanian 2013 USA^31^	CO	Veterans Affairs Medical Center, Kansas City 2000–2011	153/1056	Smoking	Current versus no	4.00 (1.90–8.10)	Hiatal hernia, heart burn duration >5 years	8
Beales 2016 USA^32^	CC	The care of the Gastroenterology Unit at the Norfolk and Norwich University Hospital NR	124/238	Aspirin Statin	At least 6 months At least 6 months	0.77 (0.46–1.14) 0.62 (0.37–0.93)	Statin, aspirin+statin NSAID, aspirin+statin	7
Bu 2006 USA^33^	CC	The University of Southern California Foregut Surgery Service 1998–2000	174/274	BMI	> 30 versus <22 kg/m^2^	3.30 (1.60–6.70)	Age and gender	6
Conio 2002 Italy^5^	CC	Eight Italian Departments of Gastroenterology gathered in a study group (GOSPE) 1995–1999	149/308	Smoking Alcohol	>20 versus 0 cigarettes/day Yes versus no	0.70 (0.40–1.40) 1.30 (0.90–2.00)	Age, gender, and center	7
Dore 2016 Italy^34^	CO	A tertiary GI clinic in Sassari 2002–2013	133/5156	Smoking BMI	Current versus no >30 versus <25 kg/m^2^	0.45 (0.20–1.00) 0.97 (0.42–2.23)	GERD, *H pylori*, gender, BMI, age, and hiatal hernia	7
Edelstein 2007 USA^35^	CC	Western Washington residents 1997–2000	193/211	BMI	>30 versus <25 kg/m^2^	2.04 (1.40–2.97)	Age, sex, and cigarette	7
Edelstein 2009 USA^36^	CC	Western Washington residents 1997–2000	97/418	Smoking	Current versus no	1.30 (0.60–2.70)	Age, gender, WHR, and clinic	7
El‐Serag 2005 USA^37^	CC	MEDVAMC 2000–2003	36/93	BMI	>30 versus <25 kg/m^2^	4.00 (1.44–11.10)	NR	6
Filiberti 2015 Italy^38^	CC	Twelve endoscopic units 2009–2012	339/619	Smoking	>18 versus no cigarettes/day	1.86 (0.98–3.16)	Age, gender, BMI, alcohol, years of schooling, and duration of reflux and collaborative center	7
Gerson 2007 USA^39^	CO	Stanford University, VA Palo Alto Health Care System, University of Arizona, Tucson VA Medical Center, and California Pacific Medical Center 2000–2004	165/751	Smoking Alcohol BMI	Current versus no Yes versus no > 30 versus 18.4–24.9 kg/m^2^	1.33 (0.90–1.98) 1.06 (0.71–1.58) 1.11 (0.50–2.47)	Age, gender male, race, GERD duration, income level, alcohol, and family history	7
Goldberg 2015 USA^40^	CC	Phoenix Veterans Affairs (VA) Hospital, as well as from a separate secure database of endoscopic procedural data 2005–2009	250/250	NSAID Aspirin PPI	Yes versus no Yes versus no Yes versus no	0.71 (0.48–1.04) 0.70 (0.47–1.05) 0.53 (0.35–0.81)	NR NR Multivitamins/age/race	6
Hilal 2016 USA^41^	CC	MEDVAMC 2008–2013	307/1724	Physical activity	High versus low level	1.19 (0.82–1.73)	Age, sex, race, GERD, *H*. *pylori*, WHR, and BMI	7
Ibiebele 2013 Australia^42^	CC	Study of Digestive Health (SDH) 2003–2006	258/569	Folate	379 versus 196 µg/d	1.17 (0.70–1.96)	Age, gender, education, BMI, heartburn or acid reflux, alcohol, smoking, NSAID use, and total energy intake	7
Jacobson 2011 USA^43^	CO	Nurses’ Health Study 1980–2004	261/15861	BMI	> 30 versus <20–24.99 kg/m^2^	1.49 (1.04–2.13)	Age, physical activity, smoking, caloric intake, alcohol, postmenopausal hormone use, and history of diabetes	8
Jacobson 2011 USA^44^	CO	Nurses’ Health Study 1980–2006	377/20863	Smoking	>50 versus 0 pack‐year	1.45 (0.95–2.22)	Year of endoscopy, age, BMI, physical activity, caloric intake, alcohol, and postmenopausal hormone use	8
Jiao 2013 USA^45^	CC	MEDVAMC 2008–2011	151/777	Selenium Vitamin C Folate Fiber	60.9 versus 40.1 µg/day 73.3 versus 25.1 mg/day 316 versus 179 µg/day 11.0 versus 5.84 g/day	0.95 (0.62–1.46) 0.79 (0.47–1.34) 0.52 (0.30–0.67) 0.50 (0.28–0.90)	Age, energy intake, sex, ethnicity, smoking, alcohol, WHR, aspirin, PPI, GERD, and physical activity	7
Jiao 2013 USA^46^	CC	MEDVAMC 2008–2011	151/777	Total meat	Tertile	1.61 (0.82–3.16)	Age, energy, sex, ethnicity, smoking, alcohol, WHR, aspirin, PPI, GERD, physical activity, dark‐green vegetables, and CML‐AGEs	7
Johansson 2007 Sweden^47^	CS	Two hospitals in southeastern Sweden (Kalmar and Vaxjo) 1997–1999	21/498	Smoking Alcohol BMI	Ever versus never Yes versus no >26.6 versus <23.6 kg/m^2^	1.80 (0.70–4.40) 0.60 (0.20–1.70) 1.10 (0.30–3.30)	Age, gender, reflux symptoms, BMI, alcohol, and *H pylori*	7
Jonaitis 2011 Lithuania^48^	CC	The Republican Panevėžys Hospital NR	33/4032	Smoking BMI	>10 versus no cigarettes/day 29.33 versus 27.54 kg/m^2^	4.62 (1.01–12.51) 1.11 (0.92–1.33)	Age, hiatal hernia, gender, BMI, *H*. *pylori*, and ulcer/stricture of esophagus	6
Keszei 2013 Netherlands^49^	CO	The Netherlands cohort study 2002–2005	447/120852	Total meat White meat	Tertile Tertile	0.79 (0.59–1.06) 0.95 (0.79–1.13)	Age, smoking, total energy intake, BMI, vegetables, fruits, education, physical activity, lower esophageal sphincter relaxing medications, and alcohol	9
Khalaf 2014 USA^50^	CC	MEDVAMC 2008–2013	323/502	NSAID	Daily versus none	1.03 (0.78–1.37)	Age, sex, race, GERD symptoms, PPI use, WHR, and *H*. *pylori*	8
Kubo 2008 USA^9^	CC	The Kaiser Permanente, Northern California 2002–2005	296/309	Selenium Vitamin C	133 versus 46 µg/day 184 versus 43 mg/day	0.58 (0.26–1.30) 0.85 (0.45–1.58)	Age, sex, race, geographic region, energy, and long‐term vitamin supplement use	7
Kubo 2009 USA^51^	CC	The Kaiser Permanente, Northern California 2002–2005	320/317	Smoking	Current versus no	1.09 (0.68–1.74)	Age, race, gender, and education	8
Kubo 2009 USA^52^	CC	The Kaiser Permanente, Northern California 2002–2005	320/317	Alcohol	14+ drinks/week versus no	1.44 (0.68–3.04)	Age, race, gender, education, smoking, *H*. *pylori*, BMI, income, and location of diagnosis	8
Kubo 2009 USA^53^	CC	The Kaiser Permanente, Northern California 2002–2005	296/309	Fiber Total meat	29.7 versus 8.6 g/day Quartile	0.95 (0.93–0.98) 0.83 (0.66–1.04)	Age, sex, race, long‐term vitamin use, and energy intake	7
Kulig 2004 Germany, Austria, and Switzerland^54^	CO	The Progression of GERD (ProGERD) study 2002–2005	702/6215	Smoking Alcohol BMI Physical activity PPI	Current versus no >0.1151 mean vol/week versus none > 30 versus 18.5–24.9 kg/m^2^ Physical versus sitting Previous intake versus no	1.65 (1.28–2.12) 1.27 (0.97–1.66) 1.04 (1.02–1.07) 0.89 (0.45–1.79) 1.57 (1.31–1.90)	Age, gender, BMI, duration of disease, PPI use, and education	9
Kuo 2010 China^55^	CC	Chang Gung Memorial Hospital Feb–Oct 2007	13/736	Smoking Alcohol	Current versus no Yes versus no	0.70 (0.20–3.30) 3.00 (0.40–25.50)	NR	6
Lam 2008 USA^56^	CS	An outpatient community‐based gastroenterology practice in northern California 2000–2006	56/280	Smoking Alcohol	Current versus no Yes versus no	1.71 (0.78–3.76) 1.29 (0.58–2.86)	Age, sex, ethnicity, and alcohol	7
Leggett 2013 USA^57^	CC	The Mayo Clinic and the Olmsted Medical Center Institutional Review Boards 1999–2006	103/103	Smoking Alcohol	Ever versus never > 7 versus <7 drinks/day	1.10 (0.60–2.10) 2.00 (0.50–8.00)	NR	7
Mathew 2011 India^58^	CO	The gastroenterology outpatient department services of King Edward Memorial Hospital 2006–2008	46/278	Smoking Alcohol BMI	Ever versus never Yes versus no > 25 versus ≤25 kg/m^2^	1.40 (0.70–2.82) 0.88 (0.32–2.43) 1.12 (0.56–2.24)	NR	6
Matsuzaki 2015 Japan^59^	CC	Keio University Hospital 2012–2013	139/2469	Smoking Alcohol Sleep time PPI	Current versus no >40 g/day versus no <6 versus >6 h/night Yes versus no	1.37 (0.83–2.26) 1.71 (1.14–2.56) 1.73 (1.21–2.46) 1.93 (1.10–3.38)	Age	6
Mulholland 2009 Northern Ireland and Republic of Ireland^60^	CC	The FINBAR study 2002–2005	224/260	Fiber	≥17.7 versus <13.7 g/day	0.40 (0.22–0.73)	Age, sex, energy intake, smoking, BMI, education, occupation, alcohol, NSAID use, location, and *H*. *pylori*	7
Murphy 2010 Northern Ireland and Republic of Ireland^61^	CC	The FINBAR study 2002–2004	220/256	Statin Vitamin C	≥72 versus <53 µg/day ≥166 versus <100 mg/day	1.08 (0.64–1.83) 0.64 (0.36–1.13)	Age, sex, BMI, energy intake, smoking, education, occupation, alcohol, NSAID use, GERD, location, and *H pylori*	7
Navab 2015 USA^62^	CS	A 600‐bed tertiary care center in the United States 1999–2008	158/442	Smoking	Current versus no	0.90 (0.82–0.99)	NR	7
Nguyen 2014 USA^63^	CC	MEDVAMC 2008–2013	301/1651	PPI	Yes versus no	1.88 (1.40–2.52)	Sex, age, race, *H*. *pylori*, WHR, active/chronic gastritis, GERD, NSAID‐only use, hiatus hernia, and statin use	7
Nguyen 2014 USA^64^	CC	MEDVAMC 2008–2013	303/909	Statin	Yes versus no	0.60 (0.39–0.93)	Age, sex, race, GERD, *H pylori*, WHR, PPI use, aspirin use, and smoking	7
O'Doherty 2011 Northern Ireland and Republic of Ireland^7^	CC	The FINBAR study 2002–2005	220/256	Total meat White meat	Quartile Quartile	0.95 (0.43–2.08) 0.56 (0.23–1.34)	Age, sex, smoking, job type, education, energy intake, fruits, vegetables, alcohol, *H pylori*, NSAID, GERD, and location	8
Omer 2012 USA^65^	CC	The Massachusetts General Hospital 1997–2010	434/434	Smoking Alcohol BMI NSAID Aspirin PPI Statin	Current versus past >14 versus <2 drinks/week > 30 versus 18.5–24.9 kg/m^2^ Current versus no Current versus no Current versus past Current versus past	1.20 (0.84–1.70) 1.10 (0.59–1.90) 1.20 (0.84–1.60) 0.92 (0.53–1.60) 0.56 (0.39–0.80) 0.91 (0.64–1.30) 0.79 (0.54–1.20)	Age, gender, race, BMI, alcohol, PPI, H2RA use, aspirin use, NSAID use, and statin use	7
Park 2009 Korea^66^	CO	Scientific Committee of the Korean College of Helicobacter and Upper Gastrointestinal Research Jan–Jul 2006	193/21832	Smoking Alcohol BMI NSAID	Current versus no Yes versus no >25 versus <23 kg/m^2^ Yes versus no	1.28 (0.88–1.85) 0.90 (0.63–1.29) 0.90 (0.63–1.29) 2.02 (1.19–3.42)	Sex, NSAID, hiatal hernia, age, BMI, cholesterol, and alcohol	7
Peng 2009 China^67^	CC	The First Affiliated Hospital of Sun‐Yat Sen University 2006–2007	27/2580	Smoking Alcohol BMI NSAID PPI	Current versus no Yes versus no >25 versus <25 kg/m^2^ Yes versus no Yes versus no	0.51 (0.07–3.96) 5.32 (1.55–13.33) 2.49 (0.66–9.43) 0.35 (0.05–2.74) 0.98 (0.97–0.98)	NR	6
Ronkainen 2005 Sweden^68^	CO	Northern Sweden, Kalix and Haparanda NR	16/1000	Smoking Alcohol	Current versus no Yes versus no	2.87 (1.01–8.13) 3.00 (1.03–8.54)	Age and sex	6
Rubenstein 2008 USA^69^	CC	Michigan Medical Center and the Ann Arbor Veterans Affairs Medical Center NR	50/50	Smoking	Current versus no	6.30 (1.90–21.00)	Adiponectin, GERD, BMI, WHR, waist circumference, and CRP	6
Schneider 2015 USA^70^	CC	The Kaiser Permanente Northern California (KPNC) 2002–2005	320/317	NSAID Aspirin	> weekly use versus no > weekly use versus no	0.89 (0.58–1.36) 0.59 (0.39–0.87)	Age, sex, race, smoking, *H*. *pylori*, ferritin, cardiovascular disease, and GERD	7
Sharp 2013 Northern Ireland and the Republic of Ireland^71^	CC	The FINBAR study 2002–2005	220/256	Folate	≥421 versus ≤318 µg/day	0.40 (0.21–0.75)	Age, sex, energy, social class, WHR, hernia, and history of gallstones	7
Shinkai 2014 Japan^72^	CC	Ten general hospitals located in the Tohoku district, the northeastern region of the main island of Japan 2010–2012	113/113	BMI PPI	> 25.0 versus <22.9 kg/m^2^ Yes versus no	3.45 (1.30–9.13) 8.21 (2.96–123.1)	Smoking, drinking, hiatal hernia, heartburn, and PPI	7
Smith 2009 Australia^73^	CC	The Queensland Institute of Medical Research and participating hospitals 2003–2006	285/644	Smoking	Current versus no	2.41 (1.39–4.17)	Age, sex, education, BMI, alcohol, aspirin, and GERD	8
Steevens 2011 Netherlands^74^	CO	The prospective Netherlands Cohort Study 1986–2002	370/120852	Smoking Alcohol BMI	Current versus no >30 g/day versus no >30 versus 18.5–25 kg/m^2^	0.93 (0.68–1.28) 1.15 (0.93–1.42) 1.48 (0.96–2.28)	Age, BMI, and alcohol	8
Stein 2005 USA^75^	CS	Southern Arizona Veteran's Affairs Healthcare System 1998–2004	65/385	BMI	> 30 versus <25 kg/m^2^	2.46 (1.11–5.44)	Age and race	7
Thota 2016 USA^76^	CO	Cleveland Clinic 2000–2012	261/1239	BMI	> 40 versus <25 kg/m^2^	1.20 (0.86–1.80)	Age, sex, and hernia size	7
Thrift 2011 Australia^77^	CC	Queensland Institute of Medical Research 2003–2006	266/585	NSAID Aspirin	> weekly use versus no > weekly use versus no	0.78 (0.46–1.31) 1.34 (0.79–2.26)	Age, gender, education, smoking, BMI, heartburn or acid reflux symptoms, and alcohol	
Thrift 2011 Australia^78^	CC	Queensland Institute of Medical Research 2003–2006	598/644	Alcohol	>42 versus <1 drink/week	0.71 (0.31–1.36)	Age, sex, education, smoking, BMI, heartburn or acid reflux symptoms, aspirin or NSAID use, and PPIs use	7
Thrift 2012 Australia^79^	CC	Queensland Institute of Medical Research 2003–2006	285/313	Physical activity PPI	High versus low index Ever versus never	0.95 (0.63–1.43) 2.07 (1.46–2.93)	Sex, education, BMI, smoking, alcohol, H2Rs or PPIs, NSAIDs, fruits, and vegetables	7
Thrift 2014 USA^80^	CC	MEDVAMC 2008–2012	711/1145	Smoking Alcohol	Current versus no Current versus no	1.07 (0.79–1.45) 1.06 (0.78–1.44)	Age, race, GERD, WHR, *H*. *pylori*, PPI, and NSAIDs	7
Thrift 2014 Western Europe, Australia, and North America^81^	CC	The Barrett's and Esophageal Adenocarcinoma Genetic Susceptibility Study (BEAGESS) 1992–2010	2061/2169	BMI	>30 versus <25 kg/m^2^	1.04 (1.03–1.06)	Age, sex, education, smoking, GERD, acid suppressant medication use, and NSAID use	8
Tseng 2008 China^82^	CO	The National Taiwan University Hospital 2003–2006	11/16647	Physical activity Sleep time	5 times versus twice/week <5 versus >8 h/day	1.48 (0.42–5.20) 2.65 (0.40–17.56)	NR	6
Veugelers 2006 Canada^83^	CC	The QEII Health Science Center (QEII HSC), Halifax 2001–2003	130/102	Smoking Alcohol BMI Vitamin C Fiber	>5000 lps versus no >40 versus <1 drink/month > 30 versus 18.5–25 kg/m^2^ ≥132 versus <132 mg/day ≥22 versus <22 g/day	1.38 (0.78–2.45) 1.68 (1.00–2.82) 2.09 (0.95–4.58) 0.44 (0.20–0.98) 0.41 (0.19–0.88)	Age and gender	7
Yates 2014 UK^84^	CO	European Prospective Investigation of Cancer‐Norfolk study 1997–2008	104/23670	Smoking Alcohol BMI	Current versus no > 28 units versus 0 > 35 versus <18.5–23 kg/m^2^	1.57 (0.83–2.96) 0.84 (0.34–2.10) 3.21 (0.59–17.57)	Age and gender	7

BMI, body mass index (kg/m^2^); CC, case–control; CML‐AGEs, Nε‐(carboxymethyl) lysine‐Advanced glycation end‐products; CO, cohort; CRP, C‐reactive protein; CS, cross‐sectional; FINBAR, Factors Influencing the Barrett's Adenocarcinoma Relationship; GERD, gastroesophageal reflux disease; lps, lifetime packs of cigarettes; MEDVAMC, Michael E. DeBakey Veterans Affairs Medical Center; NR, not reported; NSAID, nonsteroidal anti‐inflammatory drug; PPI, proton pump inhibitor; WHR, waist‐to‐hip ratio.

### 3.2 Smoking

#### 3.2.1 Current versus never

**FIGURE 1:**
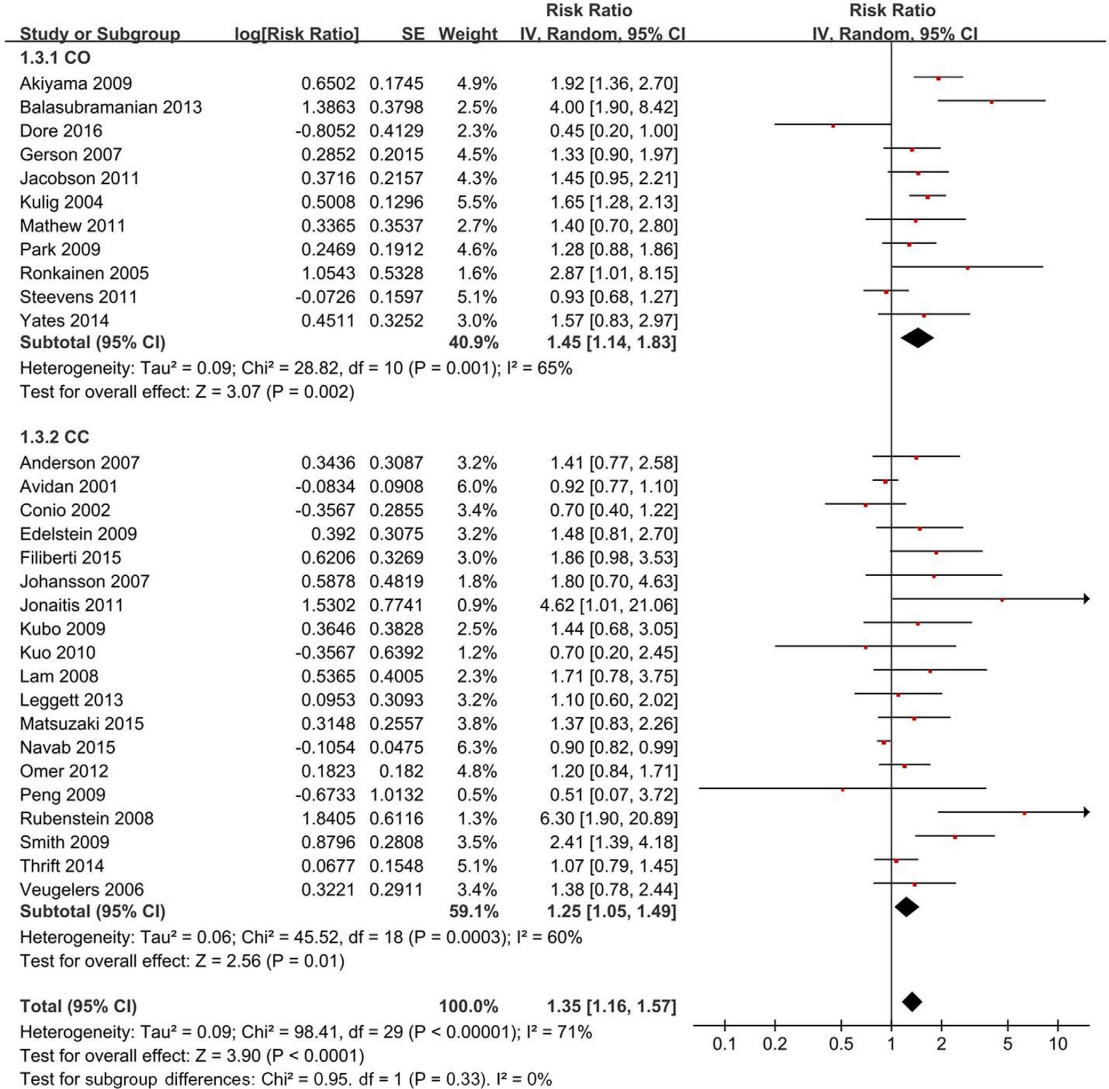
Forest plot of smoking (current vs. never) and Barrett's esophagus risk. The results demonstrated that smoking is associated with Barrett's esophagus risk

Thirty studies that involved 225,250 participants were included and a random‐effects model yielded a positive association (RR = 1.35, 95% CI = 1.16–1.57) (Figure 1). Additionally, the association was unchanged by the separate evaluations based on the study design, with 1.45 (1.14–1.83) for cohort studies and 1.25 (1.05–1.49) for case–control studies (Figure 1, Table 2).

**TABLE 2:** Subgroup analyses of smoking (current vs. never) and Barrett's esophagus risk

	n	RR (95% CI)	*P*_o_	*P*_s_	*I_s_^2^* (%)	*P*_h_	*I_h_^2^* (%)
All studies	30	**1.35 (1.16–1.57)**	**< 0.01**	< 0.01	71		
Study design							
CO	11	**1.45 (1.14–1.83)**	**<0.01**	<0.01	65		
CC‐CS	19	**1.25 (1.05–1.49)**	**0.01**	<0.01	60	0.33	0
Geographic area							
Europe	10	1.31 (0.96–1.80)	0.09	<0.01	66		
America	13	**1.28 (1.06–1.55)**	**0.01**	<0.01	70		
Asia–Australia	7	**1.55 (1.21–1.98)**	**<0.01**	0.24	25	0.47	0
Sample size							
≥200	11	**1.36 (1.11–1.66)**	**<0.01**	<0.01	71		
<200	19	1.36 (1.08–1.71)	0.01	<0.01	68	1	0
Publication year							
2009 or later	20	**1.33 (1.10–1.61)**	**<0.01**	<0.01	71		
Before 2009	10	1.41 (1.06–1.88)	0.02	<0.01	70	0.74	0
Quality score							
High	20	**1.32 (1.12–1.55)**	**<0.01**	<0.01	65		
Low or moderate	10	1.56 (1.04–2.33)	0.03	<0.01	67	0.45	0
Adjusted variables Alcohol							
Yes	12	**1.40 (1.15–1.70)**	**<0.01**	<0.01	68		
No	18	1.31 (1.04–1.65)	0.02	<0.01	67	0.67	0
BMI							
Yes	16	**1.52 (1.23–1.88)**	**<0.01**	<0.01	66		
No	14	1.10 (0.94–1.28)	0.23	0.06	40	0.01	83.3
Reflux symptom							
Yes	12	**1.56 (1.19–2.03)**	**<0.01**	<0.01	64		
No	18	1.22 (1.03–1.43)	0.02	<0.01	62	0.12	59

Note

Boldface indicates statistical significance.

CO, cohort; CC, case–control; CS, cross‐sectional; BMI, body mass index; *P*_o_, test for overall effect; *P*_s_, *P* value for heterogeneity within each subgroup. *P*_h_, *P* value for heterogeneity between subgroups. *I_s_^2^*, *I^2^* value for heterogeneity within each subgroup. *I_h_^2^*, *I^2^* value for heterogeneity between subgroups.

#### 3.2.2 Former versus never

Eleven studies met the criteria, and a significant increased BE risk was observed (RR = 1.37, 95% CI = 1.16–1.62) (Figure S2A). The changes in the recalculated RRs were not significant, with a range from 1.29 (1.10–1.50) when excluding Smith 2009 (8.1%) to 1.45 (1.18–1.78) when excluding Navab 2015 (14.9%).

#### 3.2.3 Highest versus lowest category

Four studies were included in the analysis for the highest to lowest number of cigarettes/day (Figure S2B), and a fixed‐effects model yielded a significantly positive association (RR = 1.36, 95% CI = 1.02–1.81) without heterogeneity (*p* = 0.72, *I^2^* = 0%). The dose–response analysis of the number of cigarettes/day was not conducted due to the limited studies.

#### 3.2.4 Dose–response analysis

We conducted a dose–response analysis to further explore the association between pack‐years of smoking and BE risk. Six studies were included, and the results of 1.10 (1.05–1.14) indicated that BE risk increases by 10% for each 10‐year increment. We further checked for nonlinearity of the dose–response association, and the evidence suggested that the best‐fitting model was a nonlinear model (*P*_nonlinearity_ < 0.01, Figure S3A).

#### 3.2.5 Heterogeneity

There was significant heterogeneity (*p* < 0.01, *I^2^* = 71%), but subgroup analyses (Table 2) for highest versus lowest exposure suggested that the positive association was stable by all of the confounders (study design, geographic area, publication year, sample size, quality score, alcohol, BMI, and reflux symptom).

#### 3.2.6 Publication bias

**FIGURE 2:**
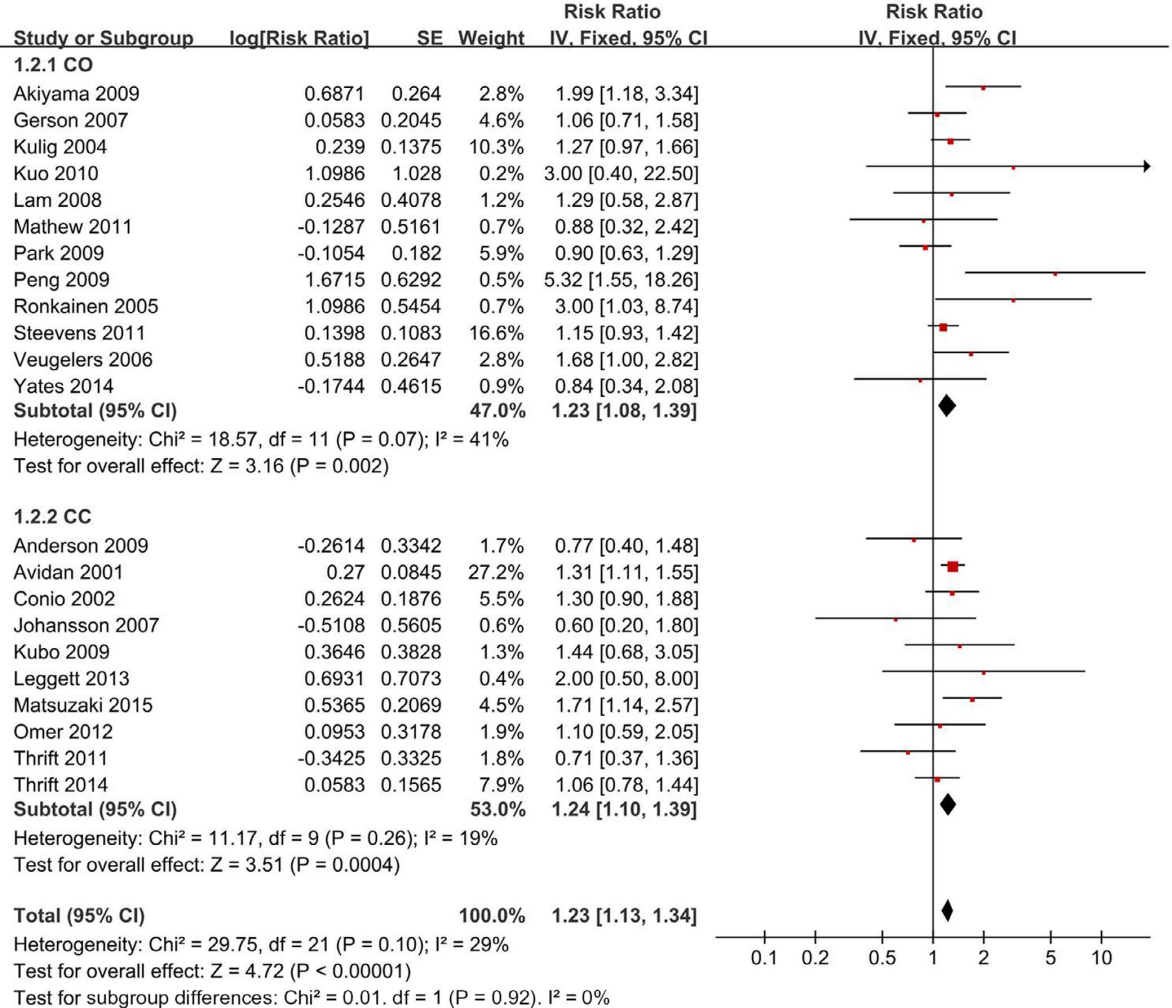
Forest plot of alcohol intake (highest vs. lowest category) and Barrett's esophagus risk. The results demonstrated that higher alcohol intake is associated with Barrett's esophagus risk

A funnel plot, Begg's test, and Egger's test were used to explore the publication bias. Indeed, Egger's test indicated evidence of publication bias (*p* < 0.1), but the funnel plot provided a visible result of no publication bias observed in Figure S3A. Additionally, the changes in the recalculated RRs were not significant (Figure 2), with a range from 1.32 (1.14–1.53) when excluding Akiyama 2009 (4.9%) to 1.38 (1.19–1.61) when excluding Navab 2015 (6.3%).

### 3.3 Alcohol

#### 3.3.1 Highest versus lowest intake

Twenty‐two studies that involved 191,725 participants were included, and a fixed‐effects model yielded a positive association (RR = 1.23, 95% CI = 1.13–1.34) (Figure 2). Additionally, the association was unchanged in cohort studies (RR = 1.23, 95% CI = 1.08–1.39) and case–control studies (RR = 1.24, 95% CI = 1.10–1.39) (Figure 2, Table 3). Nonlinear dose–response analysis could not be conducted due to the limited studies.

**TABLE 3:** Subgroup analyses of alcohol intake (highest vs. lowest category) and Barrett's esophagus risk

	n	RR (95% CI)	*P*_o_	*P*_s_	*I_s_^2^* (%)	*P*_h_	*I_h_^2^* (%)
All studies	22	**1.23 (1.13–1.34)**	**<0.01**	0.10	29		
Study design							
CO	12	**1.23 (1.08–1.39)**	**<0.01**	0.07	41		
CC‐CS	10	**1.24 (1.10–1.39)**	**0.01**	0.26	19	0.92	0
Geographic area							
Europe	7	**1.18 (1.02–1.36)**	**<0.01**	0.31	16		
America	8	**1.25 (1.11–1.42)**	**0.02**	0.77	0		
Asia–Australia	7	**1.28 (1.04–1.59)**	**0.02**	<0.01	67	0.76	0
Sample size							
≥200	9	**1.22 (1.10–1.35)**	**<0.01**	0.25	21		
<200	13	1.27 (1.08–1.49)	<0.01	0.08	38	69	0
Publication year							
2009 or later	14	**1.16 (1.03–1.32)**	**0.02**	0.05	41		
Before 2009	8	1.29 (1.15–1.46)	<0.01	0.51	0	0.23	31.3
Quality score							
High	15	**1.20 (1.10–1.31)**	**<0.01**	0.31	13		
Low or moderate	7	1.45 (1.14–1.86)	<0.01	0.10	43	0.15	51.7
Adjusted variables Smoking							
Yes	12	**1.21 (1.09–1.34)**	**<0.01**	0.11	34		
No	10	1.29 (1.10–1.51)	<0.01	0.19	28	0.50	0
BMI							
Yes	10	**1.17 (1.04–1.32)**	**<0.01**	0.10	39		
No	12	1.30 (1.14–1.47)	<0.01	0.25	20	0.26	21.5
Reflux symptom							
Yes	9	**1.19 (1.01–1.39)**	**0.04**	0.20	28		
No	13	1.25 (1.13–1.38)	<0.01	0.10	35	0.60	0

Note

Boldface indicates statistical significance.

CO, cohort; CC, case–control; CS, cross‐sectional; BMI, body mass index; *P*_o_, test for overall effect; *P*_s_, *P* value for heterogeneity within each subgroup. *P*_h_, *P* value for heterogeneity between subgroups. *I_s_^2^*, *I^2^* value for heterogeneity within each subgroup. *I_h_^2^*, *I^2^* value for heterogeneity between subgroups.

#### 3.3.2 Heterogeneity

There was no significant heterogeneity (*p* < 0.10, *I^2^* = 29%) and subgroup analyses also suggested that the positive association was stable by all of the confounders (Table 3).

#### 3.3.3 Publication bias

The funnel plot (Figure S4B) and Egger's test (*p* = 0.34) suggested no significant evidence of publication bias. Additionally, the sensitivity analysis also showed that the changes in the recalculated RRs were not significant, with a range from 1.20 (1.09–1.33) when excluding Avidan 2001 (27.2%) to 1.26 (1.15–1.37) when excluding Park 2009 (5.9%).

### 3.4 BMI

#### 3.4.1 Highest versus lowest category

**FIGURE 3:**
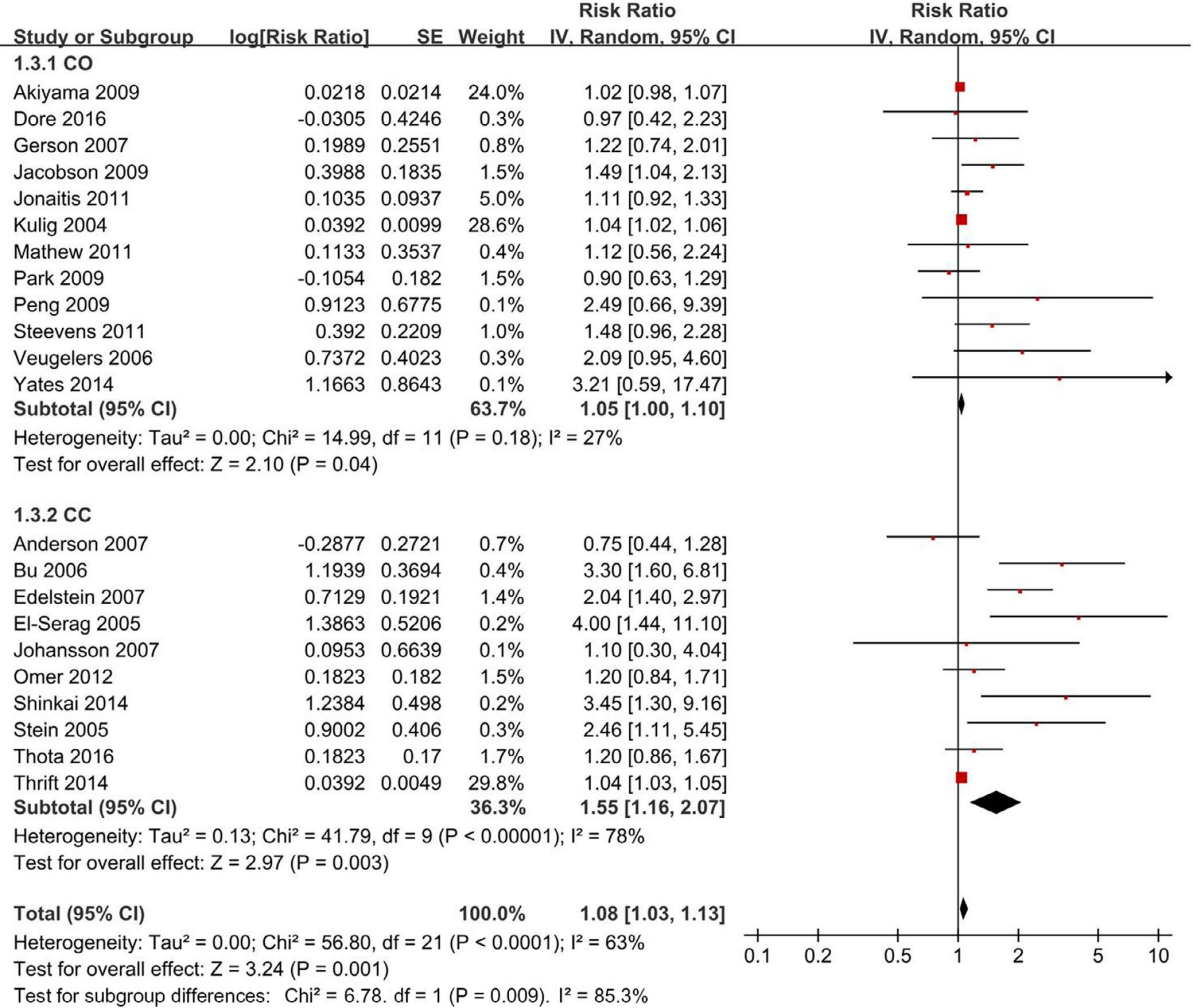
Forest plot of BMI (highest vs. lowest category) and Barrett's esophagus risk. The results demonstrated that high BMI is associated with Barrett's esophagus risk

Twenty‐two studies that involved 211,607 participants were included, and a random‐effects model yielded a positive association (RR = 1.08, 95% CI = 1.03–1.13) (Figure 3). The association was unchanged by the separate evaluations based on study design (Table 4).

**TABLE 4:** Subgroup analyses of BMI (highest vs. lowest category) and Barrett's esophagus risk.

	n	RR (95% CI)	*P*_o_	*P*_s_	*I_s_^2^* (%)	*P*_h_	*I_h_^2^* (%)
All studies	22	**1.08 (1.03–1.13)**	**<0.01**	<0.01	63		
Study design							
CO	12	**1.05 (1.00–1.10)**	**0.04**	0.18	27		
CC‐CS	10	**1.55 (1.16–2.07)**	**<0.01**	<0.01	78	< 0.01	85.3
Geographic area							
Europe	8	**1.04 (1.03–1.05)**	**<0.01**	0.52	0		
America	9	**1.69 (1.32–2.18)**	**0.02**	0.04	51		
Asia	5	1.14 (0.85–1.53)	0.38	0.08	51	< 0.01	86.5
Sample size							
≥200	8	**1.04 (1.02–1.06)**	**<0.01**	0.20	29		
<200	14	1.63 (1.25–2.14)	<0.01	<0.01	62	< 0.01	90.7
Publication year							
2009 or later	13	**1.06 (1.01–1.12)**	**0.03**	0.10	36		
Before 2009	9	1.62 (1.14–2.31)	<0.01	<0.01	79	0.02	81.5
Quality score							
High	15	**1.05 (1.02–1.09)**	**<0.01**	<0.01	58		
Low or moderate	7	1.84 (1.15–2.93)	0.01	<0.01	66	0.02	81.7
Adjusted variables Smoking							
Yes	12	**1.09 (1.01–1.17)**	**0.02**	<0.01	61		
No	10	1.62 (1.20–2.20)	<0.01	<0.01	68	0.01	84.2
Alcohol							
Yes	8	**1.04 (1.00–1.08)**	**0.03**	0.28	19		
No	14	1.48 (1.19–1.84)	<0.01	<0.01	73	< 0.01	89.8
Reflux symptom							
Yes	7	**1.04 (1.02–1.06)**	**<0.01**	0.26	22		
No	15	1.41 (1.18–1.68)	<0.01	<0.01	71	< 0.01	91.1

Note

Boldface indicates statistical significance.

CO, cohort; CC, case–control; CS, cross‐sectional; BMI, body mass index; *P*_o_, test for overall effect; *P*_s_, *P* value for heterogeneity within each subgroup. *P*_h_, *P* value for heterogeneity between subgroups. *I_s_^2^*, *I^2^* value for heterogeneity within each subgroup. *I_h_^2^*, *I^2^* value for heterogeneity between subgroups.

#### 3.4.2 Dose–response analysis

Twelve studies were included, and the results of 1.08 (1.07–1.10) indicated that the BE risk increases by 8% for each 5 kg/m^2^ increase in BMI. We further checked for nonlinearity of the dose–response association, and the evidence suggested that the best‐fitting model was a nonlinear model (*P*_nonlinearity_ < 0.01, Figure S3B).

#### 3.4.3 Heterogeneity

There was significant heterogeneity (*p* < 0.01, *I^2^* = 63%), but subgroup analyses (Table 4) suggested that the positive association was stable by all of the confounders.

#### 3.4.4 Publication bias

Indeed, the funnel plot (Figure S4C) and Egger's test (*p* < 0.1) suggested significant evidence of publication bias. However, sensitivity analysis showed that the changes in the recalculated RRs were not significant, with a range from 1.06 (1.02–1.10) when excluding Edelstein 2007 (1.4%) to 1.18 (1.08–1.29) when excluding Thrift 2014 (29.8%).

Five studies investigated BE risk and aspects of waist‐to‐hip ratio (WHR), waist circumference, hip circumference, waist‐to‐thigh ratio, and visceral adiposity. These investigations suggested that high WHR,^35^, ^63^, ^85^, ^86^ waist circumference,^35^ waist‐to‐thigh ratio,^35^ and visceral adiposity^87^ are associated with the presence of BE, whereas hip circumference (gluteofemoral obesity) may decrease BE risk.^85^

### 3.5 Physical activity and sleep time

#### 3.5.1 Physical activity

**FIGURE 4:**
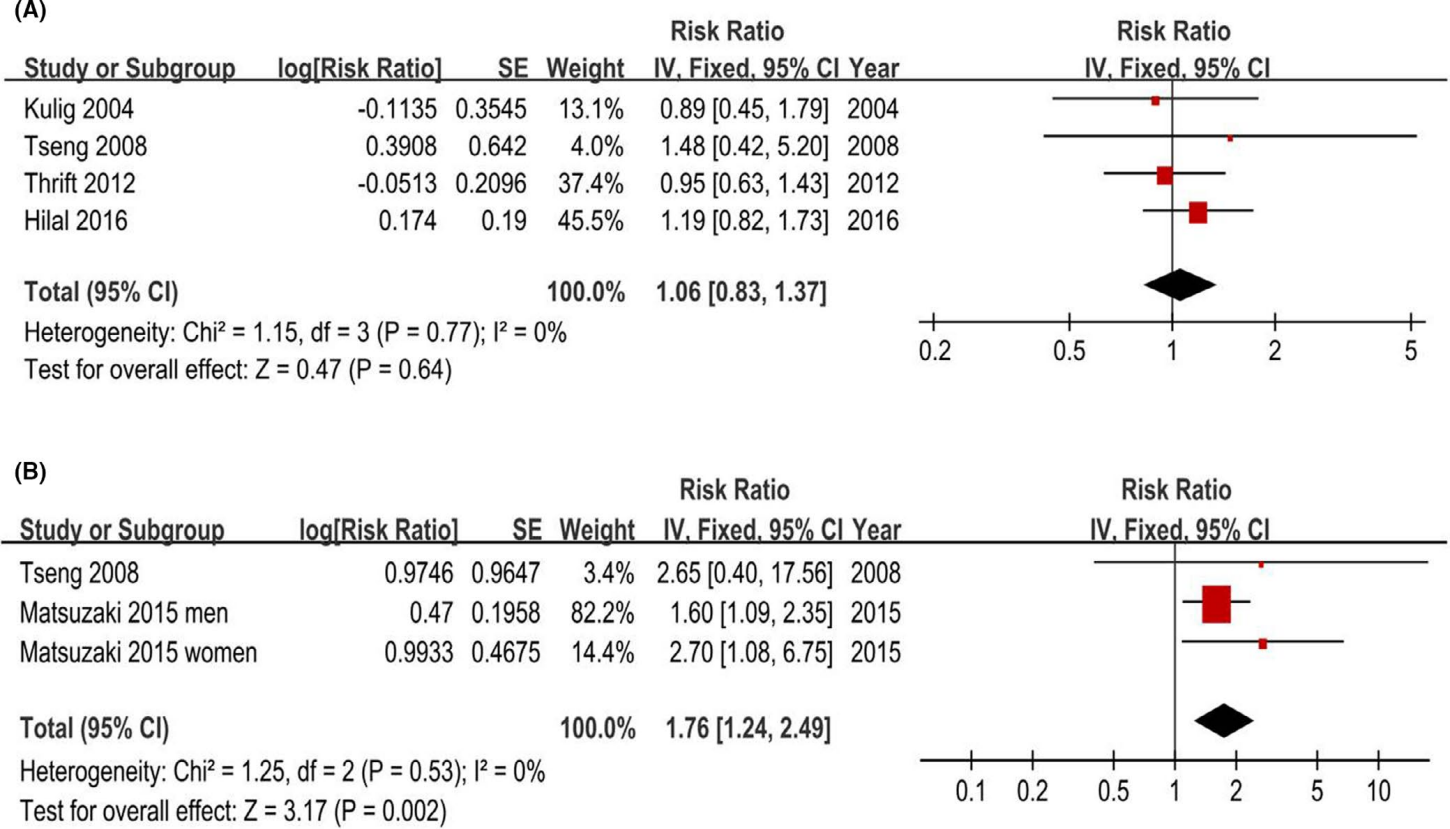
Forest plots of physical activity (highest vs. lowest category) and sleep time (<6 vs. >6 h/night) and Barrett's esophagus risk. (A) Physical activity. (B) Sleep time. The results demonstrated that longer sleep time is associated with Barrett's esophagus risk and there is no association between physical activity and Barrett's esophagus risk

Four studies were included and involved 25,491 participants. A fixed‐effects model yielded a null association (RR = 1.06, 95% CI = 0.83–1.37) without heterogeneity (*p* = 0.77, *I^2^* = 0%) (Figure 4A). Additionally, the changes in the recalculated RRs were not significant, with a range from 0.97 (0.69–1.36) to 1.14 (0.83–1.56).

#### 3.5.2 Sleep time

Two included studies that involved 2,953 participants provided 3 estimates for the sleep time<6 h a night, and a fixed‐effects model yielded a positive association (RR = 1.76, 95% CI = 1.24–2.49) without heterogeneity (*p* = 0.53, *I^2^* = 0%) (Figure 4B). The changes in the recalculated RRs also were significantly stable (Figure 6), with a range from 1.63 (1.12–2.38) to 2.69 (1.18–6.14).

### 3.6 Medications

#### 3.6.1 NSAIDs

**FIGURE 5:**
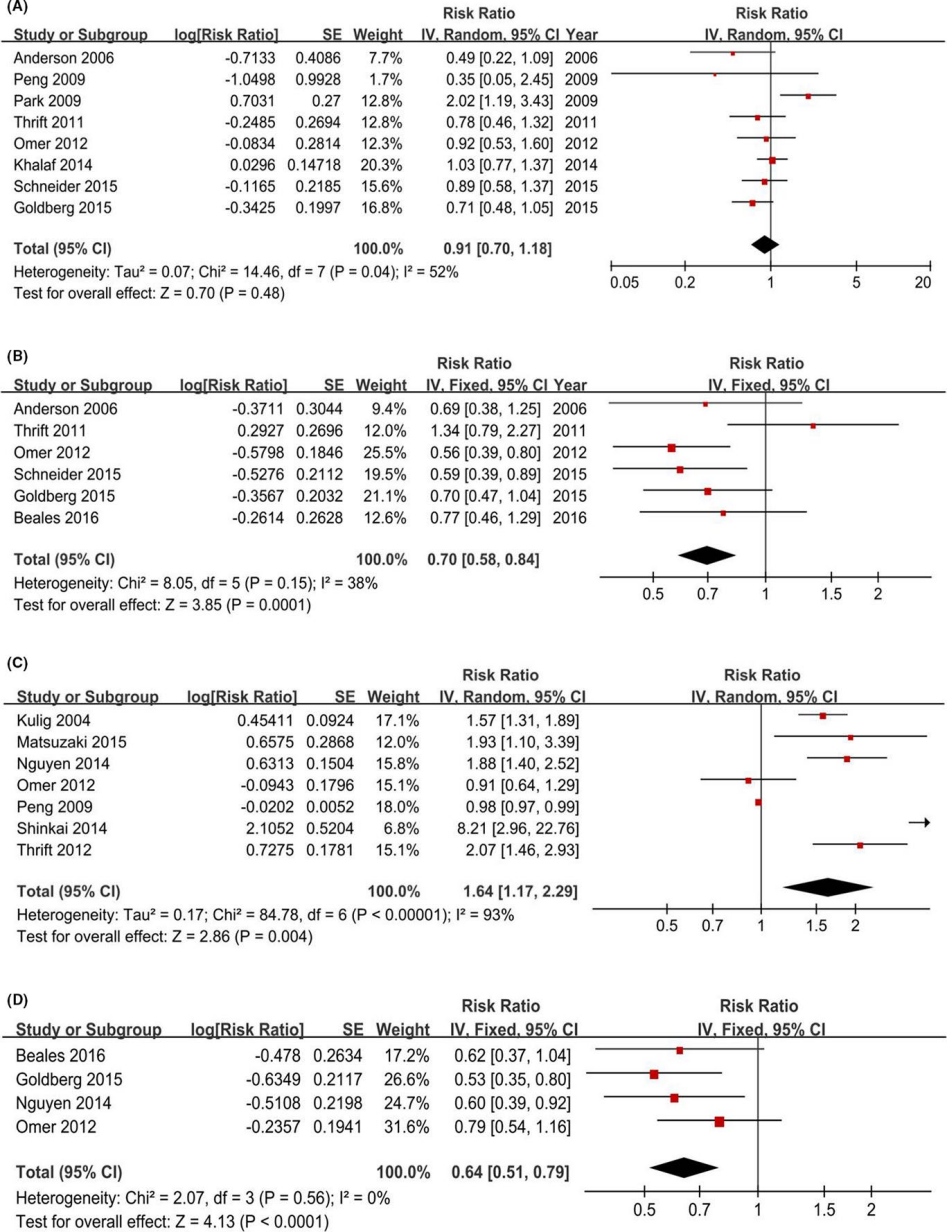
Forest plots of medications use (highest vs. lowest category) and Barrett's esophagus risk. (A) NSAIDs. (B) Aspirin. (C) PPIs. (D) Statins. The results demonstrated that aspirin intake may reduce the Barrett's esophagus risk and there is no association between NSAID, PPIs, Statins, and the risk of Barrett's esophagus

Eight studies were included that involved 28,577 participants. A random‐effects model yielded a negative association (RR = 0.91, 95% CI = 0.70–1.18) with heterogeneity (*p* = 0.04, *I^2^* = 52%) (Figure 5A). Additionally, the sensitivity analysis indicated that the changes in the recalculated RRs were not significant, with a range from 0.86 (0.72–1.02) to 0.96 (0.71–1.28).

#### 3.6.2 Aspirin

Six studies were included that involved 3,742 participants. A fixed‐effects model yielded an inversed association (RR = 0.70, 95% CI = 0.58–0.84) (Figure 5B). The changes in the recalculated RRs were significant stable, with a range from 0.64 (0.53–0.78) to 0.75 (0.61–0.93).

#### 3.6.3 PPIs

Seven studies were included that involved 14,908 participants. A significant increased BE risk was observed (RR = 1.64, 95% CI = 1.17–2.29) (Figure 5C). The sensitivity analysis indicated no evidence of publication bias, with a range from 1.45 (1.05–2.00) to 1.84 (1.24–2.72).

#### 3.6.4 Statins

Four studies were included that involved 4,845 participants. A significant reduced BE risk was observed (RR = 0.64, 95% CI = 0.51–0.79) (Figure 5D). The sensitivity analysis indicated no evidence of publication bias, with a range from 0.58 (0.45–0.75) to 0.68 (0.53–0.87).

### 3.7 Dietary factors

**FIGURE 6:**
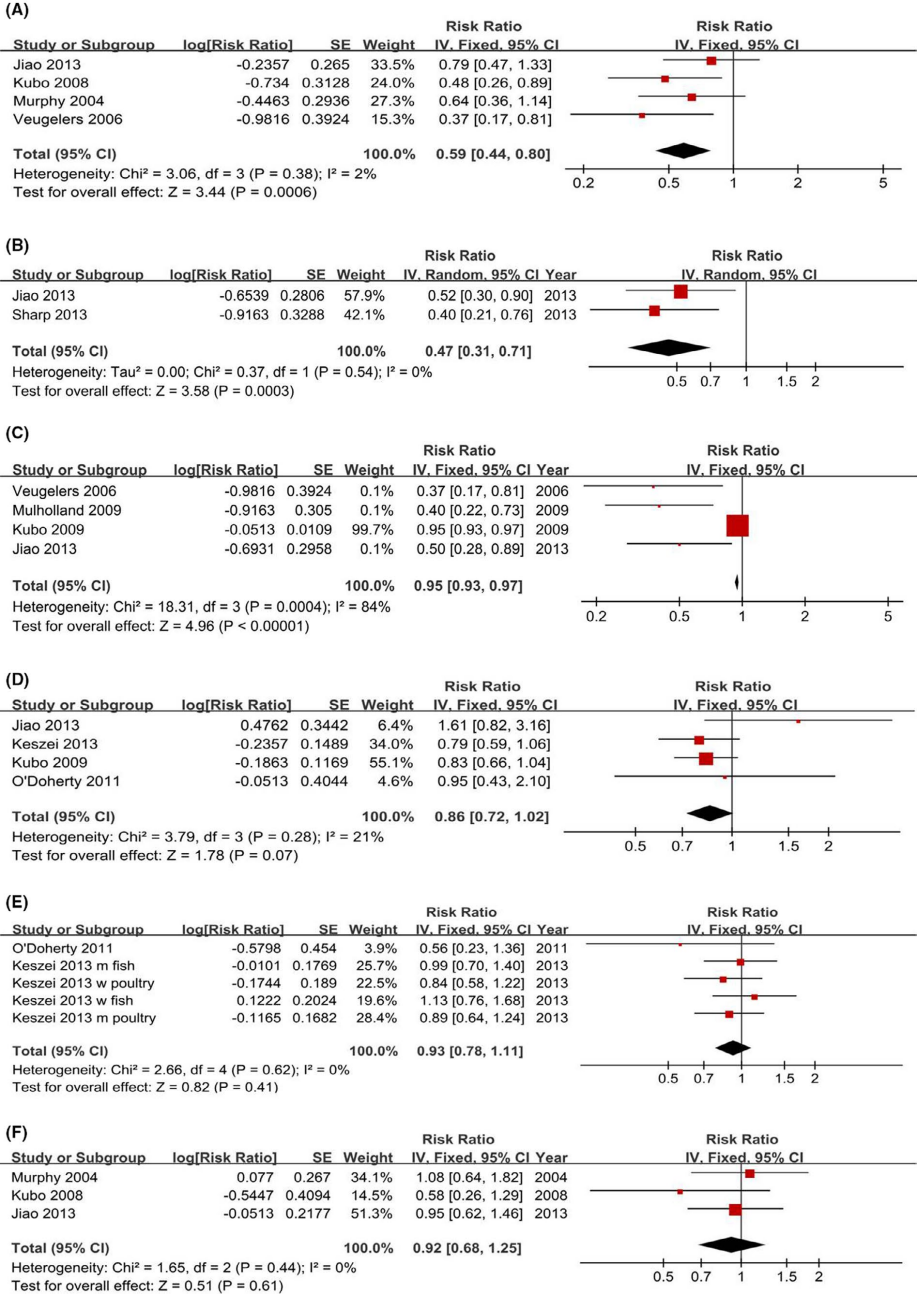
Forest plots of dietary intakes (highest vs. lowest category) and Barrett's esophagus risk. (A) Vitamin C. (B) Folate. (C) Fiber. (D): Total meat. (E) White meat. (F) Selenium. The results demonstrated that the intake of vitamin C, folate, and dietary fiber may reduce the Barrett's esophagus risk and there is no association between total meat and white meat and the risk of Barrett's esophagus

Significant inverse associations were observed between BE risks and the highest versus lowest intakes of vitamin C (4 studies involving 2,241 participants, RR = 0.59, 95% CI = 0.44–0.80, Figure 6A), folate (2 studies involving 1,404 participants, RR = 0.47, 95% CI = 0.31–0.71, Figure 6B), and dietary fiber (4 studies involving 2,261 participants, RR = 0.95, 95% CI = 0.93–0.97, Figure 6C). No associations were detected between BE risks and the highest versus lowest intakes of total meat (4 studies involving 122,861 participants, RR = 0.86, 95% CI = 0.72–1.02, Figure 6D), white meat (2 studies involving 121,328 participants, RR = 0.93, 95% CI = 0.78–1.11, Figure 6E), and selenium (3 studies involving 2,009 participants, RR = 0.92, 95% CI = 0.68–1.25, Figure 6F).

Our previous study^21^ has investigated BE risk and the intake of fruits, vegetables, fat, red meat, and processed meat, and the results demonstrated that vegetable intake was significantly associated with a decreased risk of BE, and there were no associations between the intake of fruits, fat, red meat or processed meat and BE risk. Other studies investigated the associations between the intake of vitamin B6,^42^ vitamin B12,^42^ calcium,^40^ tea,^88^ and coffee.^88^ Most of studies reported nonsignificant associations, but one study showed a decreased risk of BE with calcium intake.^40^

## 4 DISCUSSION

This large systematic analysis is the first to comprehensively explore the influence of lifestyle interventions on the risk of BE. Our analyses demonstrated a significantly increased BE risk associated with smoking, alcohol intake, high BMI, less sleep time, and PPI use. Inversed associations were observed with aspirin use, vitamin C intake, and dietary fiber intake. No associations were found for physical activity, NSAID use, folate, total meat, white meat, and selenium. Additionally, the results of detailed subgroup analyses and dose–response analyses were consistent with the original analyses.

Our analyses for smoking revealed a statistically significant 35% increased risk of BE for the current versus never smoking. When former versus never smoking was further analyzed, this increased to a 37% increased risk. In addition, the positive associations were supported by the detailed subgroup analyses. Furthermore, the dose–response analysis indicated that the BE risk increases by 10% for each 10 pack‐year increment. Additionally, the analysis for the highest to lowest number of cigarettes/day and BE risk also showed a significantly positive association. Taken together, all the analyses suggested that smoking (including current, past, longer pack‐years, and more number of cigarettes/day) may be an independent risk factor for BE development. Nevertheless, long‐term smoking cessation may diminish this risk,^31^ which suggested a feasible option for smoking cessation as a risk modification strategy. In addition, because smoking is also a well‐established risk factor for esophageal cancer,^89^, ^90^ it would be beneficial to quit smoking whenever possible, to reduce the risks of BE and esophageal adenocarcinoma.

A significant 23% increased risk was observed for highest versus lowest alcohol intake and BE risk, which was supported by the results of detailed subgroup analyses. Moreover, the International Agency for Research on Cancer (IARC, http://monographs.iarc.fr/ENG/Classification/ClassificationsGroupOrder.pdf) and the World Cancer Research Fund International (WCRF, http://wcrf.org/int/research‐we‐fund/continuous‐update‐project‐findings‐reports/summary‐global‐evidence‐cancer) have classified alcohol as a Group 1 carcinogen for esophageal cancer. Thus, a decreased intake of alcohol is advisable to reduce the risk of BE and esophageal adenocarcinoma.

Although Qumseya et al^91^ conducted a meta‐analysis to the association between obesity and BE, there were no detailed subgroup analyses, sensitivity analysis, and dose–response analysis. In our pooled analyses, body fatness was indicated by the BMI. The analyses for highest versus lowest BMI demonstrated a statistically increased risk of BE, which was supported by the results of detailed subgroup analyses. The dose–response analysis indicated that the risk was 8% for increase of per 5 kg/m^2^. Our results revealed that BMI may be an independent, strong predictor of BE. Other measures, including WHR, waist circumference, waist‐to‐thigh ratio, and visceral adiposity were also associated with increased BE risk. It remains unclear how high body fatness increases BE risk. Abdominal obesity may increase the abdominal pressure, subsequently inducing relaxation of the lower esophageal sphincter, which results in an increased risk of gastroesophageal reflux disease and thus BE.^13^, ^92^ Additionally, the continuous update report published in 2016 of WCRF on esophageal cancer has judged the evidence for the role of body fatness to be “convincing” (http://wcrf.org/int/research‐we‐fund/continuous‐update‐project‐findings‐reports/oesophageal‐cancer). Thus, keeping the weight as low as possible within the healthy range is helpful to reduce the risks of BE and esophageal adenocarcinoma.

Physical activity is often considered an inverse factor for esophageal cancer.^93^ However, our analysis revealed no association between BE risk and the highest versus lowest category of physical activity. Given the limited included studies, more studies are necessary to further verify this association. Although Lam et al^94^ conducted a meta‐analysis and found the similar results, there was only one included study and the conclusions should be considered with caution. Analyses for sleep time yielded a significant 76% increased risk of developing BE for less than 6 h a night of sleep time. Murase et al^95^ reported that short sleep time may be correlated with the severity of GERD. Nevertheless, due to the limited studies and the unclear mechanism, further studies would be helpful to clarify this association.

It is surprising that aspirin use was protective against BE with a 30% lower risk, but that nonaspirin NSAID use was not. The results were consistent with the results of the largest study^65^ to date that addressed aspirin/NSAID effect on BE. The exact mechanisms of this difference are still unclear. It is possible that the cases (more likely to be obese and having GERD) may take nonaspirin NSAIDs as a substitute for aspirin due to milder on the stomach. Analyses for PPI use yielded a statistically significant 64% increased risk of BE. PPIs are used to eradicate *H pylori* infection in combination with antibiotics, and positive *H pylori* infection is associated with a reduced risk of BE.^96^, ^97^ In contrast, the positive association may result from the fact that, in routine care, more BE patients take antacid medications such as PPIs to alleviate GERD symptoms, compared with controls.^8^, ^72^ We did not obtain the details from each study, and the positive association may thus be caused by confounding. Although the included studies for statins were limited, the present study suggested that statins may prevent BE development.

A 41% decreased risk was observed for the highest versus lowest intake of vitamin C and BE risk. Protective associations were also observed with the intake of folate and dietary fiber. Analyses for the highest versus lowest intakes of total meat, white meat, and selenium yielded nonsignificant risks of BE. Systematic analyses could not be conducted for other common dietary factors, such as other vitamins, calcium, tea, and coffee, because of the limited studies, and further studies are required to further validate our findings and to reveal these uncertain conclusions.

Our study has several strengths. The first strength was that our systematic analysis was based on the main modifiable lifestyle factors, the substantial sample size and the quantitative synthesis of the eligible data, which provided sufficient robust and reliable evidence and increased the statistical power of our findings. Second, we performed detailed subgroup analyses and dose–response analyses to further detect the associations rather than simply conducting categorical comparisons. These independent analyses provided accurate evaluations and strengthened the conclusion. Third, we broadly and systematically searched three large databases to identify studies published from inception through 30 September 2020, and the reference lists of the included studies were also searched manually to identify additional literature. Two reviewers selected the studies and extracted the data independently and in duplicate, which increased the validity of our analyses. Last, 62 included studies were identified from 16 countries or regions in the Americas, Europe, Asia, and Australia, which increased the generalizability of our results.

However, the limitations of the present meta‐analysis should be taken into consideration. First, the diagnostic criteria of BE may vary among the included studies.^17^ The updated American College of Gastroenterology (ACG) clinical guidelines recommend that intestinal metaplasia (IM) is required for the diagnosis of BE^98^ because IM is the only type of esophageal columnar epithelium that clearly predisposes to malignancy.^18^ However, this contrasts with the current British Society of Gastroenterology guidelines for BE diagnosis, which stated that IM is not necessary for the diagnosis.^19^ Second, although most of studies were adjusted for major confounders, information on some other confounders (e.g., hot drinks, *H* *pylori* infection, and hiatal hernia) could still not be obtained in several studies. Thus, our results should be considered carefully due to possible confounding. Third, the ranges of the highest to lowest category varied in the included studies, which influenced the accuracy of the results to some extent, and we cannot thoroughly exclude the potential bias. Nevertheless, to reduce the bias to a large extent, the pooled results for the highest compared with lowest category were adopted and pooled, and the results were further verified by dose–response analyses, which yielded results similar to the original analyses. Finally, the language of the included studies was limited to English, which may lead to potential selection bias.

## 5 CONCLUSIONS

This large systematic analysis demonstrated that smoking, alcohol intake, high BMI, and less sleep time are associated with BE risk. There are statistically significant reduced risks of BE with aspirin use and the intake of vitamin C, folate, and dietary fiber. Our findings strengthen our understanding of the potential mechanisms of BE development and highlight an awareness that lifestyle interventions may reduce the risks of BE and, consequently, esophageal adenocarcinoma.

## CONFLICT OF INTEREST

The authors declare no potential conflict of interest.

## AUTHOR CONTRIBUTIONS

Zhanwei Zhao and Zifang Yin wrote the main manuscript and participated in the study design and the data analysis. Chaojun Zhang completed the design of the work. All the authors have reviewed the manuscript text. ZZ and ZY contributed equally to this work.

## Supporting information

Fig S1‐S4

## Data Availability

The datasets used and analyzed during the current study are available from the corresponding author upon reasonable request.
