# Supplementary material for: Lifestyle interventions can reduce the risk of Barrett’s esophagus: a systematic review and meta‐analysis of 62 studies involving 250,157 participants
Source: Cancer Med. 2021 Jun 15;10(15):5297–320. doi: 10.1002/cam4.4061 (PMC8335822; doi:10.1002/cam4.4061)
Supplement: Supplementary file 1 — Fig S1‐S4 [file CAM4-10-5297-s001.docx]

**Figure 1.** Flowchart of the process for the identification of relevant studies.

**
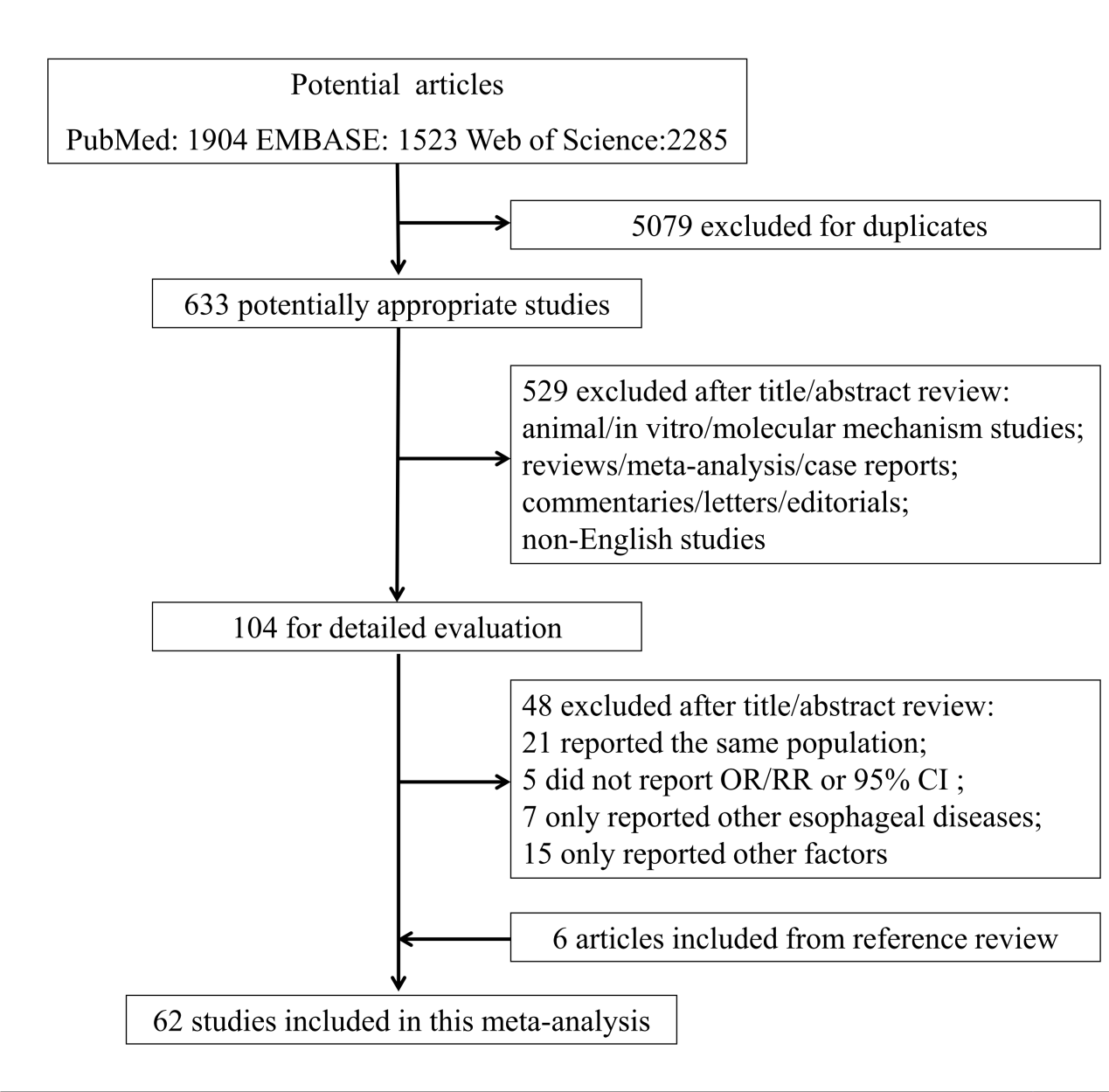
**

**Figure 2.** Forest plots of smoking (former vs never) and Barrett’s esophagus risk.

**A**

**
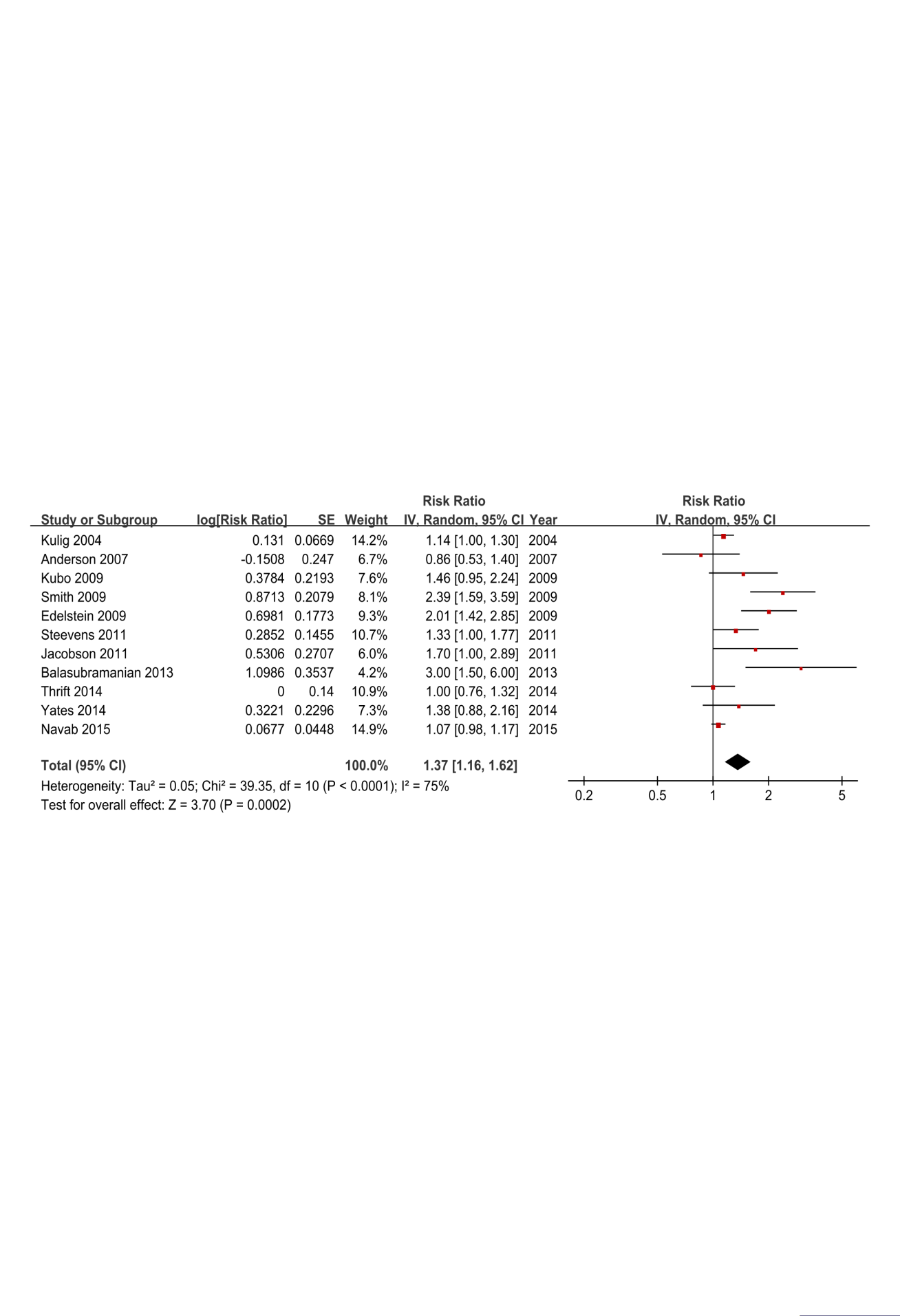
**

**B**

**
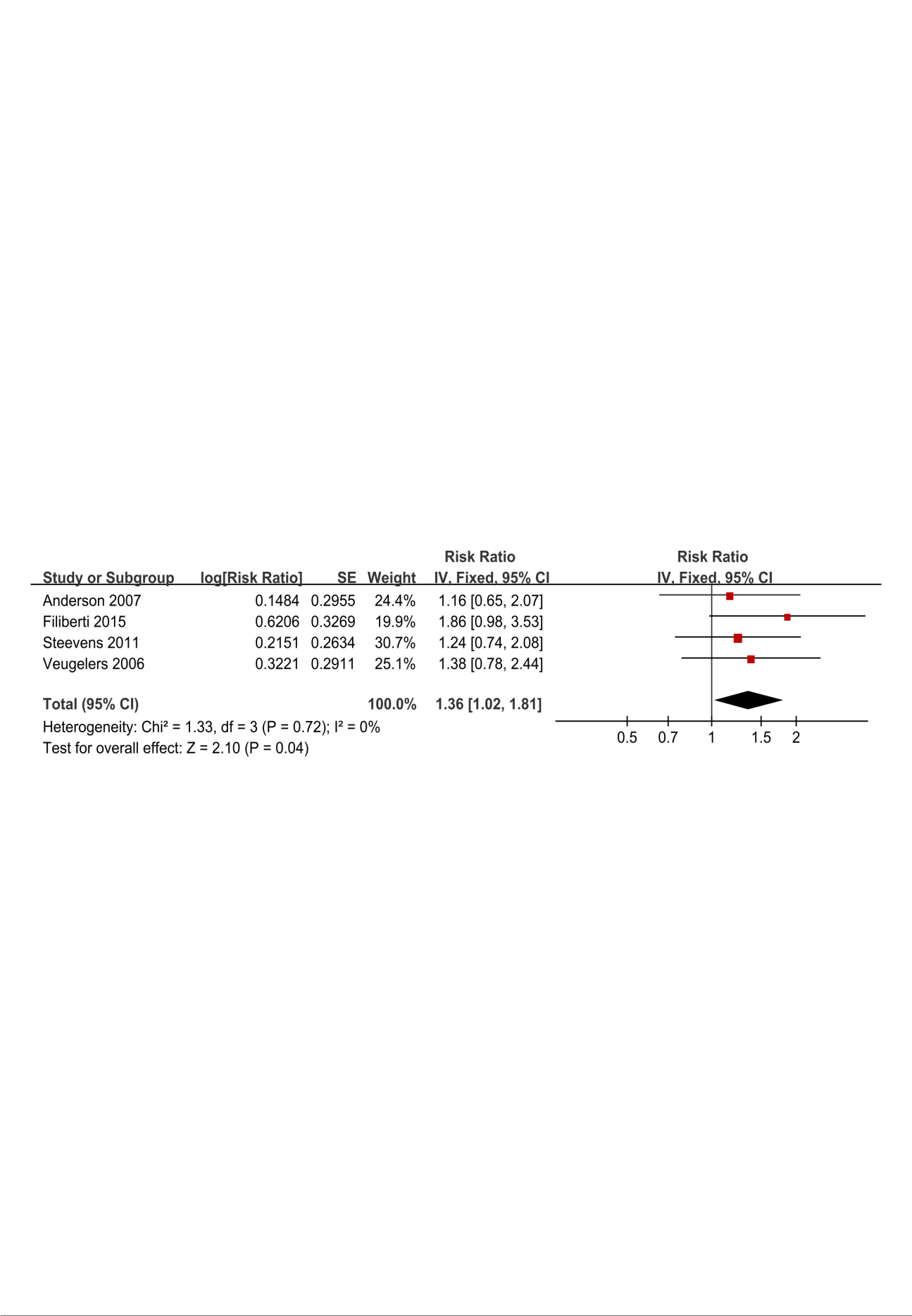
**

**Figure 3.** Nonlinear associations between (**A**) pack-years of smoking and (**B**) BMI and Barrett’s esophagus risk.

**A**

**B**

**Figure 4.** Funnel plots evaluating publication bias. **A**: Smoking. **B**: Alcohol. **C**: BMI. SE: standard error; RR: relative risk. CO: cohort studies. CC: case-control and cross-sectional studies.

**A**

**
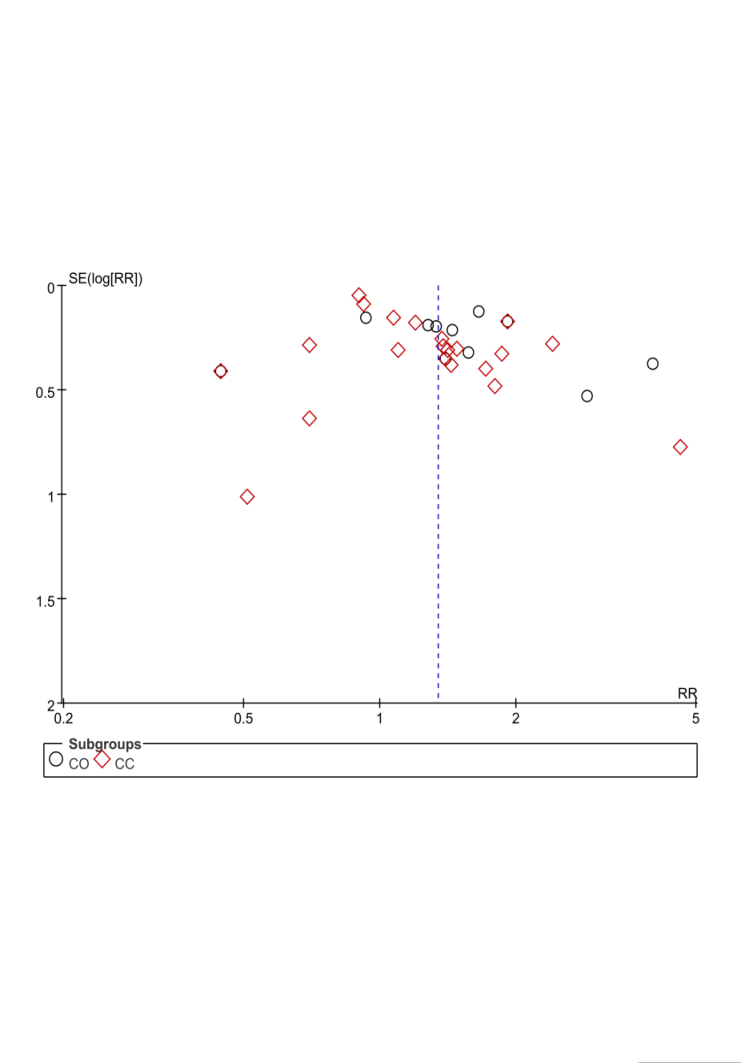
**

**B**

**
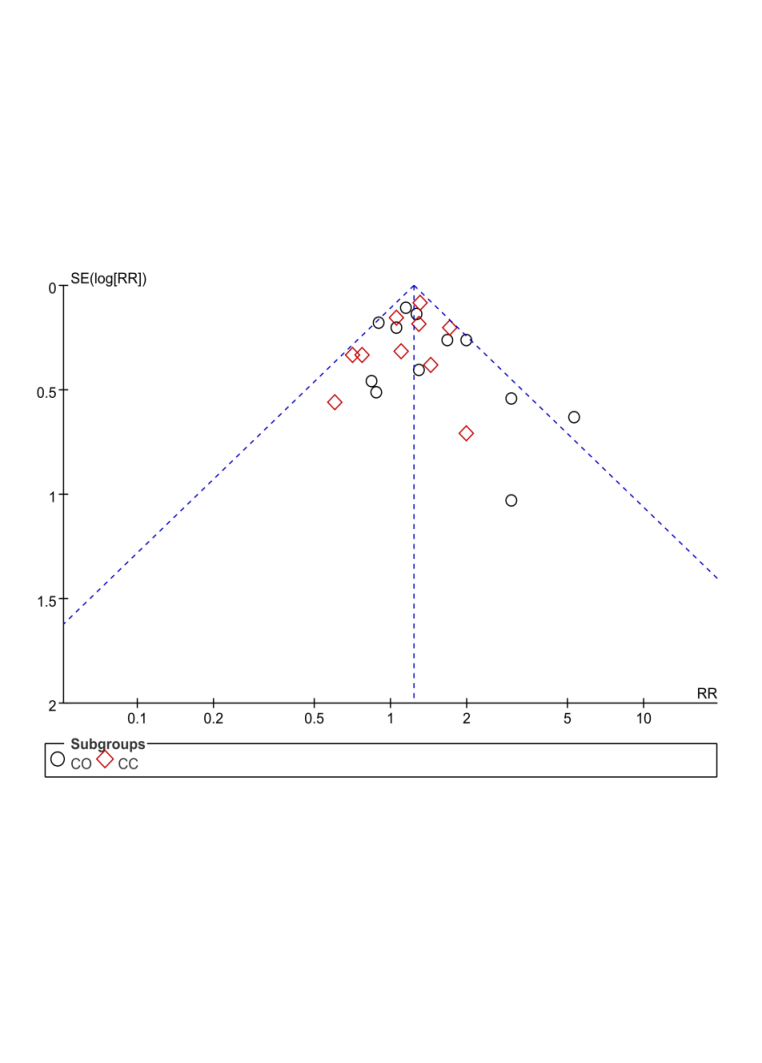
**

**C**

**
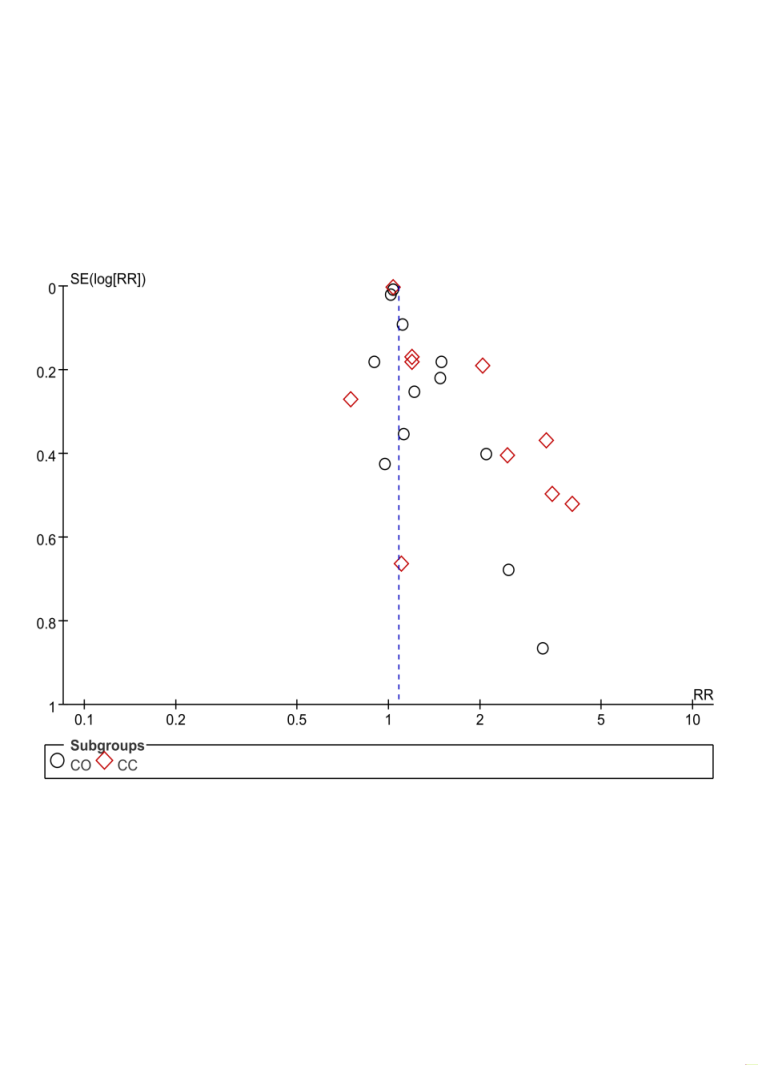
**
